# Healthy eating score and all-cause mortality: prospective findings from the Chilean National Health Survey

**DOI:** 10.1017/S0007114525104212

**Published:** 2025-08-28

**Authors:** Fabian Lanuza, Felipe Díaz-Toro, Gabriela Nazar, Yeny Concha-Cisternas, Miquel Martorell, Solange Parra-Soto, Nicole Lasserre-Laso, Tomas Meroño, Fanny Petermann-Rocha, Carlos Celis-Morales, ELHOC Research Consortium

**Affiliations:** 1 Departamento de Procesos Diagnósticos y Evaluación, Facultad de Ciencias de la Salud, Universidad Católica de Temuco, Temuco 4813302, Chile; 2 Universidad Andrés Bello, Facultad de Enfermería, Santiago 7550196, Chile; 3 Departamento de Psicología, Facultad de Ciencias Sociales, Universidad de Concepción, Concepción 4030000, Chile; 4 Centro de Vida Saludable, Universidad de Concepción, Concepción 4030000, Chile; 5 Escuela de Kinesiología, Facultad de Salud, Universidad Santo Tomás, Talca 8370003, Chile; 6 Pedagogía en Educación Física, Facultad de Educación, Universidad Autónoma de Chile, Talca 7500912, Chile; 7 Departamento de Nutrición y Dietética, Facultad de Farmacia, Universidad de Concepción, Concepción 4030000, Chile; 8 Department of Nutrition and Public Health, Faculty of Health and Food Science, Universidad del Bío-Bío, Chillan 3780000, Chile; 9 Escuela de Nutrición y Dietética, Facultad de Salud, Universidad Santo Tomás, Los Ángeles 4440000, Chile; 10 Biomarkers and Nutrimetabolomics Laboratory, Department of Nutrition, Food Sciences and Gastronomy, Faculty of Pharmacy and Food Sciences, University of Barcelona, Barcelona 08028, Spain; 11 Centro de Investigación Biomédica en Red de Fragilidad y Envejecimiento Saludable (CIBERFES), Instituto de Salud Carlos III, Madrid 28029, Spain; 12 Centro de Investigación Biomédica, Facultad de Medicina, Universidad Diego Portales, Santiago 8370068, Chile; 13 School of Cardiovascular and Metabolic Health, University of Glasgow, Glasgow G12 8QQ, UK; 14 Human Performance Lab, Education, Physical Activity and Health Research Unit, University Católica del Maule, Talca 3466706, Chile; 15 Centro de Investigación en Medicina de Altura (CEIMA), Universidad Arturo Prat, Iquique 1101214, Chile

**Keywords:** Dietary pattern, Food groups, Health, Latin American, Mortality

## Abstract

Adherence to healthy dietary patterns, including fruits, vegetables and whole grains, is linked to improved health outcomes. However, limited research has explored this association in Latin American populations. This study aimed to investigate the association between adherence to a healthy eating score (unweighted and weighted) and all-cause mortality risk in a Chilean population. This longitudinal study included 5336 Chilean participants from the Chilean National Health Survey 2016 and 2017. Six healthy eating habits were considered to produce the healthy eating score (range: 0–12): consumption of seafood, whole grains, dairy products, fruits, vegetables and legumes. A weighted score was also developed. Participants were categorised into quartiles based on their final scores, with the healthiest quartile used as the reference group. Associations between healthy eating score and all-cause mortality were performed using Cox proportional hazard models adjusted for confounders. After a median follow-up of 5·1 years, 276 (5·2 %) participants died. In the fully adjusted model, compared with participants in the healthiest quartile of the score (Q4), those in the unhealthiest quartile (Q1) had 1·61 (95 % CI: 1·14, 2·27) times higher all-cause mortality risk. A similar association was observed for the weighted healthy eating score (1·52 (95 % CI: 1·03, 2·23)). An inverse trend was observed for both scores (*P* < 0·05). Sensitivity analyses excluding participants who died within the first 2 years showed consistent results 1·63 (95 % CI: 1·09, 2·42). Individuals with the lowest healthy eating score (unweighted or weighted) had a higher mortality risk compared with their counterparts. A healthy eating score is associated with mortality risk in the Chilean population.

The global prevalence of unhealthy dietary patterns and their impact on public health have received considerable attention in recent years^(1,2)^. Poor diet quality has been consistently linked to chronic diseases, including CVD, type 2 diabetes and certain cancers, underscoring the importance of understanding how dietary adherence influences overall mortality risk^(3–5)^. Moreover, a robust body of evidence demonstrates that higher adherence to defined healthy dietary patterns – characterised by balanced intake of nutrient-dense foods and beverage groups – correlates with a reduced risk of mortality^(2,6,7)^.

However, dietary patterns and their health impacts vary by region and culture^(8,9)^, and findings from developed countries may not be fully generalisable to other contexts^(10)^. Socio-economic factors also shape dietary behaviours, as they directly affect access to healthy foods and the ability to meet dietary recommendations^(11)^.

In Latin America, where rapid urbanisation and economic disparities coexist, these factors often mediate the relationship between dietary patterns and health outcomes. Understanding this context is key to evaluating how adherence to healthy dietary patterns influences mortality, especially in populations where socio-economic inequalities may amplify barriers to achieving optimal dietary practices^(12)^. For example, a recent study conducted among elderly individuals in Costa Rica found a lower all-cause mortality associated with a traditional rural dietary pattern, where a major component was beans^(13)^.

Despite the global focus on dietary patterns, most studies have focused on regions outside of Chile, limiting the applicability of these findings to its population^(14,15)^. Chile represents a unique context characterised by distinct health determinants, dietary practices and health profiles^(16)^. Notably, 74 % of adolescents and adults over the age of 15 are classified as overweight or obese, highlighting the significant public health challenge posed by diet-related chronic conditions^(17,18)^. Although Chile’s geographical diversity provides access to a wide variety of foods, only 5 % of Chileans adhere to national dietary guidelines^(19)^.

While the associations between high diet quality and lower mortality risk are well established, most studies have been conducted in high-income countries and rely on dietary indices that may not reflect local or regional dietary patterns. In addition, conventional indices typically assign equal weight to all food groups, even though their impact on mortality risk differs considerably^(20)^.

Therefore, the aim of this study was to evaluate the association between adherence to a healthy eating score (both unweighted and weighted) and all-cause mortality risk in a nationally representative sample. This applied approach offers a more nuanced and context-specific understanding of diet quality and its public health implications.

## Methods

### Study design and participants

**Fig. 1.**
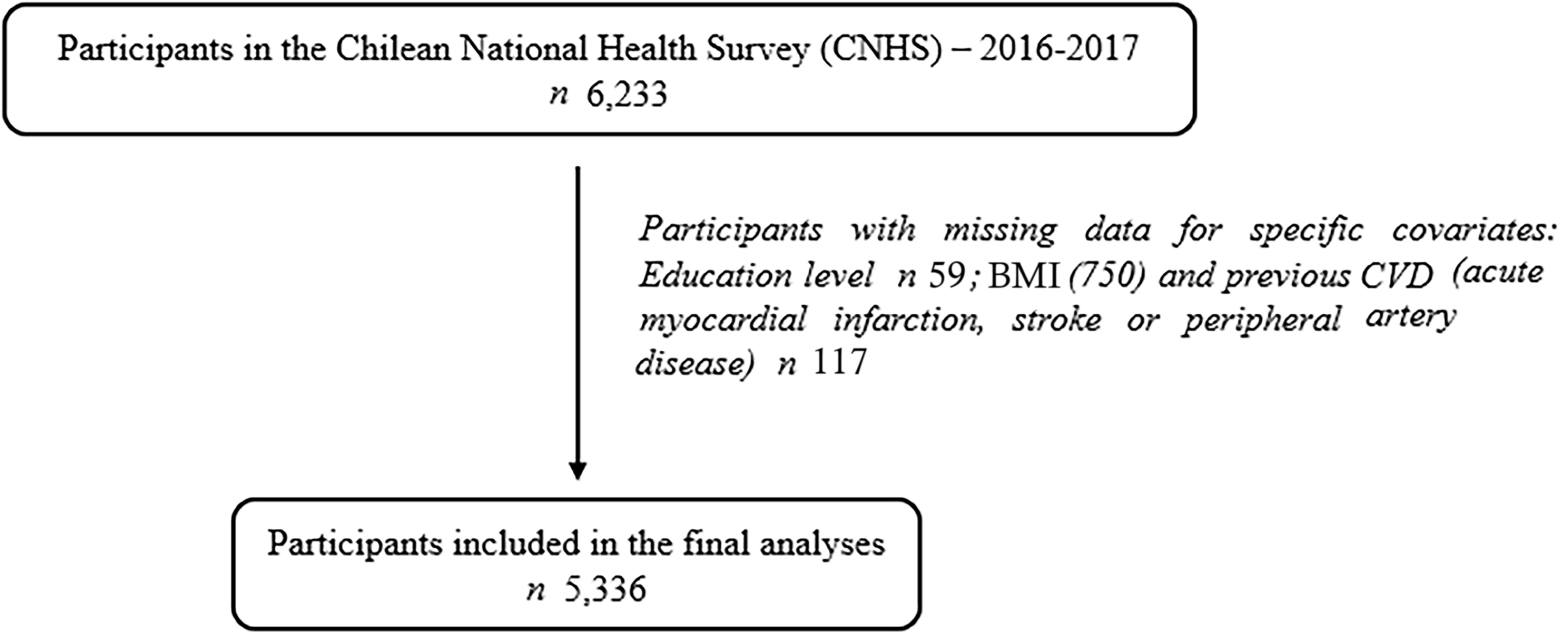
Participants included in the formal analysis. Chilean National Health Survey 2016–2017.

This prospective study included participants aged ≥ 15 years, who underwent baseline assessments during the Chilean National Health Survey 2016–2017 (CNHS 2016–2017). The CNHS 2016–2017 was a large cross-sectional, nationally representative population-based study comprising 6233 participants^(18)^. They were selected through a stratified multistage sampling of non-institutionalised individuals from urban and rural areas. Although the CNHS was originally designed as a cross-sectional survey, we conducted a prospective analysis by linking baseline data with mortality follow-up records from the Chilean Civil Registry and Identification. Trained interviewers collected data in two home visits, in which individuals were administered questionnaires (e.g. lifestyles), and measurements were taken, including anthropometric and physiological measures, as well as biological samples. Trained nurses conducted all clinical measures. From the original sample size (6233 participants), and after removing individuals with missing data on the exposure and covariates (*n* 897), the final analytical sample comprised 5336 participants (Fig. 1). Non-significant differences were observed between included and excluded groups regarding sex, geographical zone and healthy eating score (*P* > 0·05).

The CNHS 2016–2017 was funded by the Chilean Ministry of Health and led by the Department of Public Health, the Pontificia Universidad Católica de Chile. The CNHS 2016–2017 was conducted according to the guidelines laid down in the Declaration of Helsinki, and all procedures involving human subjects were approved by the Ethics Research Committee of the Faculty of Medicine at the Pontificia Universidad Católica of Chile and reviewed by the Chilean Ministry of Health. Written informed consent was obtained from all subjects (≥ 18 years) or from the caregivers of those younger than 18 years. Data are available on the Ministry of Chile webpage https://epi.minsal.cl/bases-de-datos.

### Healthy eating score (unweighted)

The healthy eating score was developed to evaluate adherence to the national dietary guidelines. It assessed the frequency of consumption of six food groups: consumption of seafood, whole grains, dairy products, fruits, vegetables and legumes. These questions were designed by experts based on the food-based dietary guidelines for the Chilean population as described in the CNHS 2009–2010^(21)^.

Frequency of consumption of seafood (How often do you consume fish and shell food?), whole grain (How often do you consume any whole-grain products like whole-grain bread, whole-grain cereal or any other food that contains whole-grain flour?), dairy products (How often do you consume milk, cheese, fresh cheese or yogurt?) and legumes (How often do you consume any type of legumes, such as beans, lentils, peas or chickpeas? The answers to these questions were scored according to the recommendations of the national dietary guidelines^(22)^, from zero point for no compliance to two points for complete compliance, according to online Supplementary Table 1. For the intake of fruits and vegetables, it was possible to calculate an average intake/d according to the dietary report of the survey. An intake of 80 g/serving was considered as one standard portion size of fruits or vegetables according to the survey guidelines of use^(18)^. Participants were scored according to tertiles of intake as g/d. Consumption of fruits and vegetables was estimated through the questions: ‘Typically, how many days a week do you eat fruits?’, ‘Typically, how many days a week do you eat vegetables or vegetable salad? (do not include legumes or potatoes)’ and ‘How many servings of fruits/vegetables or vegetable salad do you eat in one of those days? (supported by a deck of cards with standardised serving sizes)’. The healthy eating score was created, ranging from 0 to 12 points, where higher points indicated greater adherence to national dietary guidelines. This scoring method was constructed in prior research^(23)^. The score was categorised into quartiles using rank cases, with the highest quartile representing the healthiest group.

### Healthy eating score (weighted)

A weighted version of the healthy eating score was developed to account for the differential impact of individual food groups on all-cause mortality^(20)^. Each food group was scored using a binary system: 1 if the recommendation is met and 0 if it is not. Relative risks (RR) for each food group in relation to all-cause mortality were obtained from a systematic review and meta-analysis of prospective studies (online Supplementary Table 1)^(24)^.

To construct a weighted score that reflects the relative contribution of each food group to mortality risk, we applied a logarithmic transformation to normalise the RR (ln(RR)). This approach is justified by the multiplicative nature of hazard ratios (HR): a given change in RR corresponds to a proportional (rather than additive) change in risk. The logarithmic transformation linearises this relationship, making it more interpretable and suitable for constructing weights that accurately reflect the relative importance of each food group.

The weighted diet score is then calculated as a weighted sum, following the structure: Diet Score = (1 – Recommendation met) × Weight for each food group. Recommendation met is a binary variable (1 = met, 0 = not met). The individual scores for each food group are summed to derive the total score. The final score was then categorised in quartiles using rank cases, with quartile 4 representing the healthiest group. The weighted score provides a nuanced measure of dietary quality by incorporating the relative impact of different food groups on mortality risk, offering a refined tool for analysing dietary patterns.

### All-cause mortality

The outcome of the current study was all-cause mortality. The date of death was obtained at follow-up from death certificates linked to the Chilean Civil Registry and Identification. Mortality data were available until the 31st of December 2021. Therefore, mortality status was censored on this date or the date of death if this occurred earlier.

### Covariates

Sociodemographic data were collected at baseline and included age, sex, zone of residence (rural or urban), income level (low, middle and high), geographical zone (north: I–VI regions, centre: VII–IX, south: X–XV) and education level (elementary: <8 years (low), secondary: 8–12 years (middle) and higher education: ≥12 years (high)).

Health-related conditions were self-reported in response to the question: ‘Has a doctor, nurse or another health professional ever told you that you have had or currently have hypertension, high cholesterol, diabetes, peripheral artery disease or previous CVD events (e.g. myocardial infarction or stroke)?’ These long-term conditions included hypertension, hypercholesterolaemia, diabetes, peripheral artery disease and prior CVD events. They were then used to construct a multimorbidity score, categorised as follows: no long-term conditions, one long-term condition or two or more long-term conditions.

Lifestyle factors included alcohol consumption, tobacco use, sleep duration, physical activity and sedentary behaviour. Alcohol consumption was self-reported and collected using the ‘Alcohol Use Disorders Identification Test’ (AUDIT) questionnaire developed by the WHO and adapted for the Chilean population^(25)^. Tobacco status was classified as non-smoker, ex-smoker or current smoker, based on self-reported responses. Sleep duration (in h/d) was self-reported using nationally validated questionnaires. Physical activity levels, including moderate and vigorous intensities and transport-related physical activity, were determined using the Global Physical Activity Questionnaire version 2 (QPAQ v2)^(26)^. Physical activity was categorised into inactive individuals (<600 MET/min/week) and active individuals (≥600 MET/min/week)^(27)^. Sedentary behaviour was derived using the following question: ‘How much time do you usually spend sitting or reclining on a typical day?’.

Lifestyle variables were further stratified based on recommended criteria, including low-risk alcohol consumption (AUDIT score < 8 points), never smoking, adequate sleep duration (7–9 h/d), sufficient physical activity (≥ 600 MET/min/week) and low sedentary time (< 4 h/d). These criteria have been described in detail elsewhere^(15)^.

Finally, BMI was calculated as weight/height (kg/m^2^) and classified using the WHO criteria for adults (normal: 18·5–24·9 kg/m^2^; overweight: 25·0–29·9 kg/m^2^; obese: ≥ 30·0 kg/m^2^)^(28)^ and the Pan American Health Organization criteria for older adults (normal: 23·0–27·9 kg/m^2^; overweight: 28·0–31·9 kg/m2; obese: ≥ 32·0 kg/m^2^)^(29)^. Participants who were underweight were excluded due to the potential for reverse causality (*n* 197).

### Statistical analyses

Descriptive characteristics by healthy eating score quartiles are presented as means with standard deviations (sd) for continuous variables and as frequencies and percentages for categorical variables.

Crude Kaplan–Meier curves were constructed to estimate 5·1-year survival for categories of healthy eating score (quartiles). Kaplan–Meier curves were selected as they visually represent survival probabilities across quartiles, allowing for an intuitive comparison of trends over the follow-up period. In addition, sensitivity analyses were conducted in the full adjusted model, using a 2-year landmark that excluded all participants who died within the first 2 years of follow-up (*n* 79).

Associations between healthy eating score quartiles and all-cause mortality were investigated using Cox proportional hazard models. Associations between healthy eating score quartiles and all-cause mortality were investigated using Cox proportional hazard models. This method was chosen for its suitability in analysing time-to-event data while accounting for potential confounders, providing robust estimates of relative HR for all-cause mortality. Individuals in the quartile 4 (healthiest eating score) were used as reference. The results are reported as HR with their 95 % confidence intervals (95 % CI). Duration of follow-up was used as the time variable.

Analyses were adjusted for confounders based on previous literature^(30)^ using the following two models: model 1 was adjusted by age, sex, zone of residency and educational level, while model 2 was additionally adjusted for lifestyle variables (alcohol consumption, tobacco status, sleep duration, physical activity and sitting time), BMI and multimorbidity.

Finally, we also investigate whether the association between the healthy eating score (unweighted) categories and all-cause mortality differed by subgroups. We tested for interactions, and all of them were found to be non-significant in the fully adjusted model. Nevertheless, we stratified the analyses based on well-established insights from previous studies^(7)^, considering factors such as age (≥ and < 60 years), sex (men and women), zone of residence (urban and rural), geographical zone (regions) and BMI categories (online Supplementary Table 2).

The significance level was defined as *P* < 0·05. IBM SPSS 29.0 was used for statistical analyses. This study followed the STROBE reporting guidelines for cohort studies.

## Results

Over a median follow-up of 5·1 years (interquartile range: 5·0 to 5·2 years), 276 participants (5·2 %) died. Baseline characteristics according to healthy eating score quartiles are shown in Table 1. Of the total sample, 22·3 % of participants were in quartile 1 (least healthy), 31·6 % in quartile 2, 14·9 % in quartile 3 and 31·3 % in quartile 4 (most healthy) of the healthy eating score. The mean age of participants was 48·9 (19·1) years, with minimal variation across quartiles.

**Table 1.** General characteristics of the study population by quartiles of healthy eating score. Chilean National Health Survey 2016–2017

	All participants		Quartile 1		Quartile 2		Quartile 3		Quartile 4	
	*n*	%	*n*	%	*n*	%	*n*	%	*n*	%
*n* (%)	5336	100	1188	22·3	1685	31·6	795	14·9	1668	31·3
Age (years)										
Median	50		48		50		51		51	
IQR	33–64		32–63		34–64		33–66		33–64	
Sex, *n* (%)										
Women	3379	63·3	692	58·2	1062	63·0	516	64·9	1109	66·5
Men	1957	36·7	496	41·8	623	37·0	279	35·1	559	33·5
Zone of residence *n* (%)										
Urban	4480	84·0	966	81·3	1382	82·0	669	84·2	1463	87·7
Rural	856	16·0	222	18·7	303	18·0	126	15·8	205	12·3
Income level *n* (%)										
Low	2543	47·7	500	42·1	837	49·7	382	48·1	824	49·4
Middle	2113	39·6	480	40·4	643	38·2	328	41·3	662	39·7
High	680	12·7	208	17·5	205	12·2	85	10·7	182	10·9
Geographical zone (region) (%)										
North (I–VI)	1923	36·0	375	31·6	594	35·3	292	36·7	662	39·7
Centre (VII–IX)	1430	26·8	324	27·3	410	24·3	229	28·8	467	38·0
South (X–XV)	1983	37·2	489	41·2	681	40·4	274	34·5	539	32·3
Education level, *n* (%)										
Low < 8 years	1269	23·8	373	31·4	424	25·2	183	23·0	289	17·3
Middle 8–12 years	2890	54·2	640	53·9	951	56·4	428	53·8	871	52·2
High > 12 years	1177	22·1	175	14·7	310	18·4	184	23·1	508	30·5
Healthy eating score	5·3	2·2	2·3	0·8	4·5	0·5	6·0	0·1	8·0	1·1
Weighted healthy eating score	–0·53	0·1	–0·67	0·0	–0·60	0·0	–0·52	0·0	–0·37	0·1
Tobacco status (never smoking) *n* (%)	2539	47·6	510	42·9	826	49	412	51·8	791	47·7
Physical activity (active) *n* (%)	3663	68·6	748	63·0	1127	66·9	553	69·6	1235	74·0
Sleep (7–9 h/d) *n* (%)	3152	59·1	671	56·5	1000	59·3	466	58·5	1015	60·8
Sitting time (< 4 h/d) *n* (%)	1246	23·4	266	22·4	381·	22·6	197·	24·8	402	24·1
AUDIT score (< 8 pts) *n* (%)	2049	38·4	721	60·7	1013	60·1	503	63·3	1050	62·9
BMI (kg/m^2^)										
Median	28·2		28·4		28·3		28·3		28·0	
IQR	25·0–31·9		25·0–32·1		25·1–31·1		25·0–31·7		24·8–31·8	
Diabetes (yes), *n* (%)	775	14·5	141	11·9	246	14·6	115	14·5	273	16·4
Hypertension (yes), *n* (%)	1736	32·5	366	30·8	544	32·3	265	33·3	561	33·6
High cholesterol (yes), *n* (%)	1366	25·6	271	22·8	402	23·9	214	26·9	479	28·7
CVD-AMI, Stroke, (yes) *n* (%)	658	12·3	132	11·1	218	12·9	76	9·6	232	13·9

AUDIT, Alcohol Use Disorders Identification Test; AMI, acute myocardial infarction; *n*, number; IQR, interquartile range.

Notable differences were observed in education level, with a higher percentage of individuals with low education in the lowest quartile of the healthy eating score, compared with those in the highest quartile. Additionally, participants in the lower quartile were more likely to reside in rural areas and exhibit less adherence to healthy lifestyle factors, such as physical activity. Conversely, participants in the highest quartile were more likely to be women and to reside in urban areas.

**Fig. 2.**
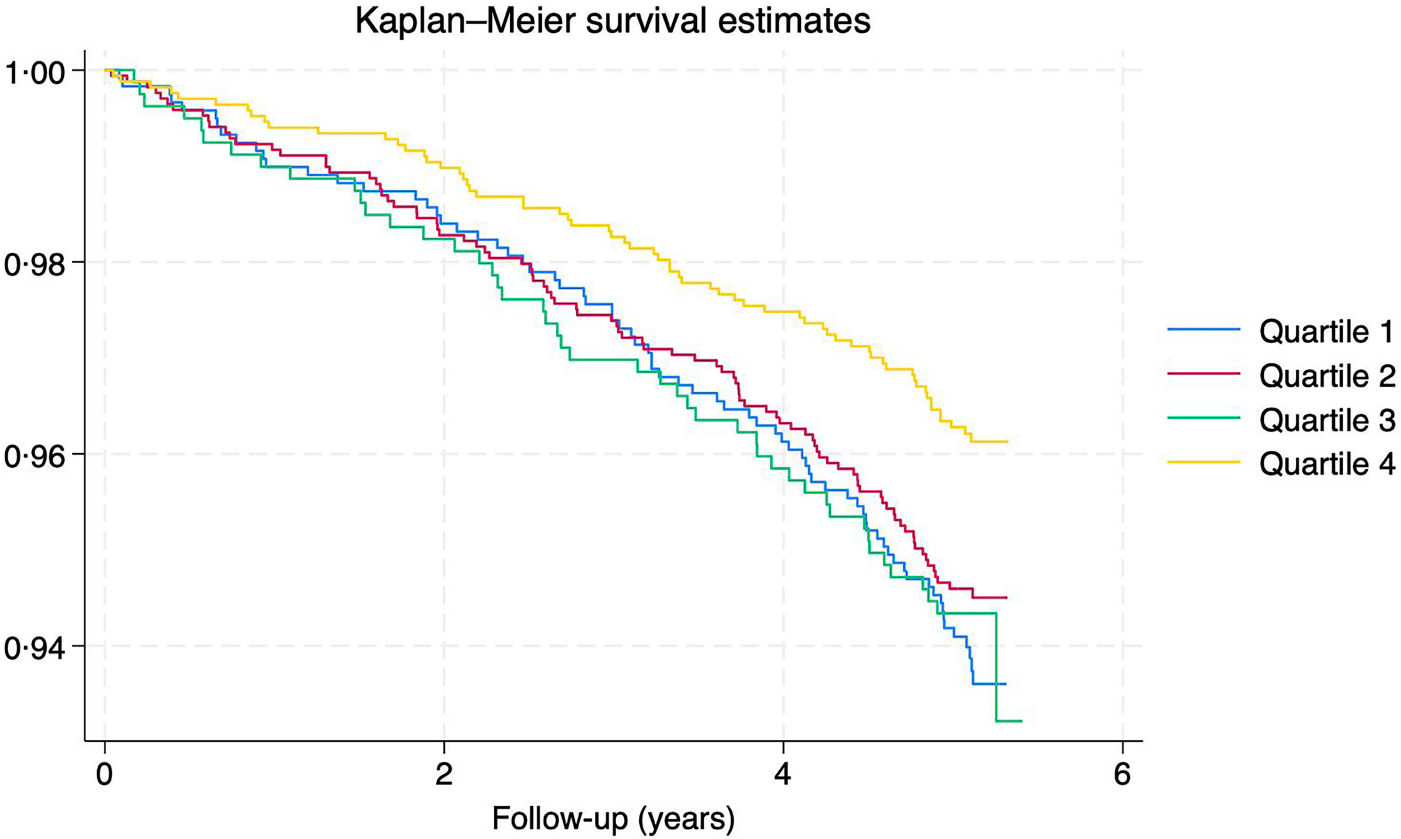
Crude Kaplan–Meier curve to estimate 5·1-year survival for healthy eating score. Chile, Chilean National Health Survey 2016–2017. Healthy eating score (Q4: highest and Q1: lowest); error bars (95 % CI).

Crude Kaplan–Meier survival estimates by healthy eating score quartiles are shown in Fig. 2. Participants in the lowest quartile of the healthy eating score had lower survival rates, followed by those in the middle quartiles (second and third). Associations between the healthy eating score (unweighted and weighted) and all-cause mortality are presented in Tables 2 and 3, respectively. In model 2, the most adjusted model, participants in the lowest quartile of the healthy eating score had a 1·61 (95 % CI: 1·14, 2·27), while those in quartile 2 and 3 had 1·44 (95 % CI: 1·04, 1·99) and 1·47 (95 % CI: 1·00, 2·16) times higher risk of mortality due to any cause, respectively, compared with those in the highest quartile of the healthy eating score. This trend persisted across all models, including the 2-year landmark analysis.

**Table 2.** Associations between unweighted healthy eating score and all-cause mortality in Chilean adults. Chilean National Health Survey 2016–2017

All-cause mortality	Total cases/ events	Ref. (Quartile 4)	Quartile 3			Quartile 2			Quartile 1			Trend		
		HR (95 % CI)	HR	95 % CI	*P*	HR	95 % CI	*P*	HR	95 % CI	*P*	HR	95 % CI	*P*
Model 1	5336/ 276	1·00 (Ref.)	1·43	0·97, 2·09	0·065	1·49	1·08, 2·06	**0·013**	1·67	1·20, 2·34	**0·002**	0·84	0·76, 0·94	**0·002**
Model 2	5336/ 276	1·00 (Ref.)	1·47	1·00, 2·16	**0·046**	1·44	1·04, 1·99	**0·025**	1·61	1·14, 2·27	**0·006**	0·86	0·77, 0·96	**0·007**
2-year landmark*	5060/ 197	1·00 (Ref.)	1·36	0·87, 2·15	0·174	1·29	0·88, 1·89	0·191	1·63	1·09, 2·42	**0·015**	0·86	0·76, 0·98	**0·024**

HR, hazard ratio. Analyses are presented as HR and their 95 % CI. Individuals in the quartile 4 were used as the referent. Model 1: adjusted by age, sex, zone of residency and educational level; model 2: as per model 1 but additionally for lifestyle variables (alcohol consumption, tobacco status, sleep duration, physical activity and sitting time), BMI and multimorbidity.

A 2-year landmark was carried out as a sensitivity analysis, excluding people who died during the first 2 years of follow-up. *Using covariates from model 2.

**Table 3.** Associations between weighted healthy eating score and all-cause mortality in Chilean adults. Chilean National Health Survey 2016–2017

All-cause mortality	Total cases/ events	Ref. (Quartile 4)	Quartile 3			Quartile 2			Quartile 1			Trend		
		HR (95 % CI)	HR	95 % CI	*P*	HR	95 % CI	*P*	HR	95 % CI	*P*	HR	95 % CI	*P*
Model 1	5336/ 276	1·00 (Ref.)	1·17	0·81, 1·69	0·378	1·14	0·79, 1·65	0·464	1·58	1·08, 2·31	**0·018**	0·87	0·77, 0·98	**< 0·031**
Model 2	5336/ 276	1·00 (Ref.)	1·13	0·78, 1·63	0·503	1·13	0·78, 1·64	0·509	1·52	1·03, 2·23	**0·033**	0·88	0·78, 0·99	**0·042**
2-year landmark*	5060/ 197	1·00 (Ref.)	0·97	0·63, 1·50	0·923	1·00	0·65, 1·54	0·982	1·57	1·01, 2·43	**0·044**	0·86	0·74, 0·99	**0·041**

HR, hazard ratio. Analyses are presented as HR and their 95 % CI. Individuals in the quartile 4 were used as the referent. Model 1: adjusted by age, sex, zone of residency and educational level; model 2: as per model 1 but additionally for lifestyle variables (alcohol consumption, tobacco status, sleep duration, physical activity and sitting time), BMI and multimorbidity.

A 2-year landmark was carried out as a sensitivity analysis, excluding people who died during the first 2 years of follow-up. *Using covariates from model 2.

Associations between the weighted healthy eating score and all-cause mortality are presented in Table 3. In model 2, the most adjusted model, participants in the lowest quartile of the healthy eating score had a 1·52 times higher risk of all-cause mortality (95 % CI: 1·03, 2·23) compared with those in the highest quartile. However, for participants in quartiles 2 and 3, the association lost statistical significance. This trend persisted in the 2-year landmark analysis.

Finally, associations between quartiles of the healthy eating score and all-cause mortality by subgroups are presented in online Supplementary Table 2. Notably, despite the absence of significant interactions, the increased mortality risk associated with lower healthy eating scores was more pronounced among participants aged 60 years and older, with those in the lowest quartile having a 1·58 (95 % CI: 1·08, 2·30) times higher mortality risk compared with those in the highest quartile. Additionally, participants from the North region exhibited higher mortality risks in relation to the lowest quartile (2·01 (95 % CI: 1·14, 3·54)). Women also showed an elevated risk in the lowest quartile, with an HR of 1·72 (95 % CI: 1·04, 2·87). Conversely, participants with a BMI below 24·9 or 27·9 had a substantially higher mortality risk in the lowest quartile (HR: 2·82 (95 % CI: 1·57, 5·06)) compared with those with higher BMI.

## Discussion

This study found that individuals with lower scores on the healthy eating score (unweighted and weighted) had a significantly higher risk of all-cause mortality compared with those with higher scores. These findings suggest that lower adherence to a healthy eating score, characterised by reduced consumption of seafood, whole grains, dairy products, fruits, vegetables and legumes, is associated with an increased risk of mortality in the Chilean population.

Previous studies often relied on dietary scores that did not account for the combined or weighted impact of individual food groups, limiting their ability to capture nuanced associations with mortality risk^(23,31)^. Our study adds novelty by applying a healthy eating score derived from the Chilean national dietary guidelines, tailored to regional dietary habits and public health concerns. Furthermore, we introduce a weighted version of the score, accounting for the differential health impact of food groups, which enhances the specificity and interpretability of the diet mortality associations.

The results of this study align with previous research demonstrating an inverse association between diet quality and mortality risk. For instance, studies conducted in various populations have reported that greater adherence to healthy dietary scores, such as the Healthy Eating Index (HEI) and the Mediterranean diet, is associated with a reduced risk of mortality^(1,32,33)^. In particular, HEI or alternative HEI include components that assess intake of nutrients of concern (e.g. fat, sodium, added sugars) and penalise consumption of certain foods. While this approach allows for detailed nutritional assessment, it assumes access to comprehensive dietary recall data. In contrast, our score is based on key food groups promoted by the Chilean dietary guidelines and does not include nutrients *per se* or penalize the intake of unhealthy foods, reflecting a pragmatic, food-based framework more feasible in a national survey.

It is important to acknowledge that scores such as HEI have undergone extensive validation across multiple international cohorts and are supported by strong predictive validity. By contrast, the healthy eating score proposed in our study – while grounded in national recommendations – has not yet been validated externally. Nonetheless, it responds to an urgent need to develop culturally adapted, context-sensitive tools for nutritional epidemiology in regions like Latin America, where the applicability of global scores may be limited not only by food availability but also by economic aspects and culinary practices, among others.

While most of these studies have been conducted in high-income countries in Europe and America, Chile presents a unique context. According to the Global Burden of Disease^(4)^, Chile is officially classified as a high-income economy. Nonetheless, it is often considered an emerging or transition economy due to its rapid economic growth, ongoing industrialisation and reliance on natural resources like copper, along with significant social inequities and developmental challenges. This study provides valuable insights into how these associations may differ across socio-economic and cultural contexts.

Interestingly, our score is quite similar than the Prospective Urban Rural Epidemiology (PURE) healthy eating pattern (except for whole grains *v*. nuts), which, in a combined analysis from eighty countries, was associated with a lower risk of mortality when comparing the lowest *v*. highest quintile of the healthy diet score (HR = 0·70; 95 % CI: 0·63, 0·77)^(33)^. Undoubtedly, both unweighted and weighted dietary scores are necessary despite the potential complexity and similar directional results (as seen in our data or the PURE study)^(33)^. This is because we recognise, and the evidence supports, that different food groups have varying impacts on mortality risk^(24,34,35)^.

From a different perspective, a recent large prospective cohort study in China found that while the total scores of three *a priori* dietary indices showed no significant associations with all-cause mortality, key components – such as greater dietary variety, a more balanced diet and better food adequacy – were associated with reduced all-cause mortality^(36)^. This suggests that, beyond cultural and regional differences in dietary patterns and food security issues, the methodological approach of evaluating overall diet indices or specific food groups and their components could present opposite, neutralised or combined effects on mortality. Additionally, varying weighting methodologies across dietary indices can influence results, as certain foods (e.g. nuts or olive oil in the Mediterranean diet or whole-grain cereals and fatty fish in the Nordic diet) are more prominent in high-income countries but may be less common in Latin America or Chile^(34)^. These variations, influenced by factors such as food security, food processing and culinary techniques, can affect both the strength and direction of the association between diet and mortality. Indeed, extreme geographical zones, such as the North (I–VI regions) and South (X–XV regions) of Chile, showed different associations with mortality risk compared with the central regions, highlighting the importance of context-specific dietary evaluations^(7,20)^.

Several biological mechanisms may explain the increased mortality risk observed among participants with lower scores on the HEI. Foods included in this index, such as fruits, vegetables, legumes and whole grains, are rich in vitamins, minerals, fibre and bioactive compounds such as phytochemicals that have been shown to have protective effects against chronic diseases, including CVD, type 2 diabetes and certain types of cancer^(37–39)^. In addition to traditional beneficial properties (e.g. antioxidant and antinflammatory, among others) linked with dietary fibre and (poly)phenols, these components have also been identified as key factors in direct microbe–host interactions and, therefore, in shaping the gut bacterial community^(40)^. However, in recent years, plant-based dietary indexes have not consistently been associated with a lower risk of mortality^(41,42)^. Indeed, the quality of these diets (healthy *v*. unhealthy) has been suggested as crucial in determining their effects on cardiometabolic diseases and the risk of all-cause mortality^(43,44)^.

High consumption of seafood and dairy products has also been associated with better cardiovascular health and reduced inflammation, which could contribute to a decreased mortality risk^(45)^. The *n*-3 PUFA have been attributed with properties such as anti-arrhythmic and anti-inflammatory effects and improved vascular function^(46)^. However, it is worth mentioning that fried fish consumption is likely associated with an increased risk of CVD events, reinforcing the importance of considering both the type of food and its matrix, as observed in the Costa Rican study, where, by contrast, beans are the main source of nutrients^(13)^. Regarding dairy products, the evidence is more controversial, and the effects may depend on the amount and the type of food: low-fat or full-fat dairy products such as milk, cheese and yogurt^(24,47,48)^. For example, a systematic review of prospective cohort studies did not find an association between dairy product consumption and CVD mortality^(49)^, while another systematic review found an inverse association between yogurt consumption and the risk of all-cause and CVD mortality^(48)^.

The interplay between socio-economic factors and dietary patterns highlights the importance of addressing social and economic inequalities as part of broader public health policies and strategies. Limited access to affordable, nutrient-dense foods and structural barriers, such as food deserts in rural or underprivileged urban areas, may exacerbate disparities in adherence to healthy dietary patterns. These challenges underline the necessity of integrating targeted policies and interventions that not only promote dietary education but also improve the affordability and availability of healthy foods, particularly for vulnerable populations. Recently, the new Dietary Guidelines for Chile have been relaunched^(50)^, which delivers ten new nutritional guidance messages, highlighting two of them: consumption of fresh food from fairs and established markets and sharing kitchen tasks, enjoying new and traditional preparations.

This study has several strengths, including using a large, nationally representative cohort, the application of both unweighted and weighted healthy eating scores and the adjustment for multiple confounding factors in the analyses. However, it also has some limitations. First, dietary information was self-reported, which may lead to recall bias or inaccurate reporting. Although several confounding factors were adjusted for, the presence of residual or unmeasured confounders cannot be entirely ruled out. Finally, the observational nature of the study limits the ability to establish causal relationships.

In addition, the relatively short follow-up period and limited number of deaths may reduce the statistical power to detect associations and increase the risk of reverse causality, despite the 2-year landmark sensitivity analysis conducted. Moreover, the absence of cause-specific mortality data limited our ability to explore diet-disease associations in greater detail.

Future research could focus on longitudinal and interventional studies with a longer duration to observe how changes in dietary patterns over time affect mortality risk. Also, it is important to consider the type and quality of the dietary pattern and its food groups or food items to avoid misunderstandings between studies because the complexities and relationships of the diet and health depend on the food processing, sources and preparations or culinary techniques, among others.

Additionally, qualitative studies could explore the barriers and facilitators to adhering to a healthy diet in different subgroups of the Chilean population. It would also be beneficial to investigate how other lifestyle behaviours, such as physical activity, alcohol consumption and smoking, interact with dietary patterns to influence health outcomes^(51)^. In conclusion, the findings of this study reinforce the importance of promoting healthy eating habits as a key strategy to improve public health and reduce the risk of mortality in the Chilean population.

## Supporting information

Lanuza et al. supplementary material Lanuza et al. supplementary material

## Supplementary material

For supplementary material/s referred to in this article, please visit https://doi.org/10.1017/S0007114525104212.

## References

[1] Morze J , Danielewicz A , Hoffmann G , et al. (2020) Diet quality as assessed by the healthy eating index, alternate healthy eating index, dietary approaches to stop hypertension score, and health outcomes: a second update of a systematic review and meta-analysis of cohort studies. J Acad Nutr Diet 120, 1998–2031.e15. 33067162 10.1016/j.jand.2020.08.076

[2] Sotos-Prieto M , Bhupathiraju SN , Mattei J , et al. (2017) Association of changes in diet quality with total and cause-specific mortality. N Engl J Med 377, 143–153. 28700845 10.1056/NEJMoa1613502 PMC5589446

[3] Nazar G , Díaz-Toro F , Petermann-Rocha F , et al. (2023) Multimorbidity and 11-year mortality in adults: a prospective analysis using the Chilean National Health Survey. Health Promot Int 38, daad176. 10.1093/heapro/daad176 38128083

[4] Murray CJL , Aravkin AY , Zheng P , et al. (2020) Global burden of 87 risk factors in 204 countries and territories, 1990–2019: a systematic analysis for the Global Burden of Disease Study 2019. Lancet 396, 1223–1249. 33069327 10.1016/S0140-6736(20)30752-2 PMC7566194

[5] Ahmad S , Moorthy MV , Lee I-M , et al. (2024) Mediterranean diet adherence and risk of all-cause mortality in women. JAMA Netw Open 7, e2414322. 38819819 10.1001/jamanetworkopen.2024.14322 PMC11143458

[6] Shan Z , Wang F , Li Y , et al. (2023) Healthy eating patterns and risk of total and cause-specific mortality. JAMA Intern Med 183, 142–153. 36622660 10.1001/jamainternmed.2022.6117 PMC9857813

[7] English LK , Ard JD , Bailey RL , et al. (2021) Evaluation of dietary patterns and all-cause mortality. JAMA Netw Open 4, e2122277. 34463743 10.1001/jamanetworkopen.2021.22277 PMC8408672

[8] Jayedi A , Soltani S , Abdolshahi A , et al. (2020) Healthy and unhealthy dietary patterns and the risk of chronic disease: an umbrella review of meta-analyses of prospective cohort studies. Br J Nutr 124, 1133–1144. 32600500 10.1017/S0007114520002330

[9] Wang VH-C , Foster V & Yi SS (2021) Are recommended dietary patterns equitable? *Public Health Nutr* 1–7. 10.1017/S1368980021004158 PMC8883773 34602107

[10] Harmon BE , Boushey CJ , Shvetsov YB , et al. (2015) Associations of key diet-quality indexes with mortality in the Multiethnic Cohort: the Dietary Patterns Methods Project. Am J Clin Nutr 101, 587–597. 25733644 10.3945/ajcn.114.090688 PMC4340063

[11] Mackenbach JD , Nelissen KGM , Dijkstra SC , et al. (2019) A systematic review on socioeconomic differences in the association between the food environment and dietary behaviors. Nutrients 11, 2215. 31540267 10.3390/nu11092215 PMC6769523

[12] Mujica-Coopman MF , Navarro-Rosenblatt D , López-Arana S , et al. (2020) Nutrition status in adult Chilean population: economic, ethnic and sex inequalities in a post-transitional country. Public Health Nutr 23, s39–s50. 32131930 10.1017/S1368980019004439 PMC10200670

[13] Yundan Z , Monica C-O , Ana B , et al. (2024) Traditional rural dietary pattern and all-cause mortality in a prospective cohort study of elderly Costa Ricans: the Costa Rican Longevity and Healthy Aging Study (CRELES). Am J Clin Nutr 120, 656–663. 38971470 10.1016/j.ajcnut.2024.06.022

[14] Schwingshackl L , Bogensberger B & Hoffmann G (2018) Diet quality as assessed by the healthy eating index, alternate healthy eating index, dietary approaches to stop hypertension score, and health outcomes: an updated systematic review and meta-analysis of cohort studies. J Acad Nutr Diet 118, 74–100.e11. 29111090 10.1016/j.jand.2017.08.024

[15] Petermann-Rocha F , Diaz-Toro F , Troncoso-Pantoja C , et al. (2024) Association between a lifestyle score and all-cause mortality: a prospective analysis of the Chilean National Health Survey 2009–2010. Public Health Nutr 27, e9. 10.1017/S1368980023002598 PMC10830369 38053402

[16] Martínez Arroyo A , Corvalán Aguilar C , Palma Molina X , et al. (2020) Dietary patterns of adolescents from the Chilean growth and obesity cohort study indicate poor dietary quality. Nutrients 12, 2083. 32674402 10.3390/nu12072083 PMC7400834

[17] Lanuza F , Morales G , Hidalgo-Rasmussen C , et al. (2022) Association between eating habits and quality of life among Chilean university students. J Am Coll Health 70, 280–286. 32343200 10.1080/07448481.2020.1741593

[18] MINSAL (Ministry of Health (Chile)) (2017) Encuesta Nacional de Salud 2016–2017 (National Health Survey 2016–2017). Chile: Ministerio de Salud, Gobierno de Chile.

[19] Lanuza F , Zamora-Ros R , Petermann-Rocha F , et al. (2023) Advances in polyphenol research from Chile: a literature review. Food Rev Int 39, 3134–3171.

[20] Burggraf C , Teuber R , Brosig S , et al. (2018) Review of a priori dietary quality indices in relation to their construction criteria. Nutr Rev 76, 747–764. 30053192 10.1093/nutrit/nuy027 PMC6130981

[21] Ministerio de Salud & Gobierno de Chile (Ministry of Health & Government of Chile) (2010) Encuesta Nacional de Salud 2009–2010 (National Health Survey 2009–2010). https://epi.minsal.cl/resultados-encuestas/ (accessed March 2024).

[22] Olivares Cortés S , Zacarías Hasbún I , González CG , et al. (2015) Design and validation of an image for dissemination and implementation of Chilean dietary guidelines. Nutr Hosp 32, 582–589. 26268085 10.3305/nh.2015.32.2.9084

[23] Lanuza F , Petermann-Rocha F , Celis-Morales C , et al. (2022) A healthy eating score is inversely associated with depression in older adults: results from the Chilean National Health Survey 2016–2017. Public Health Nutr 25, 2864–2875. 10.1017/S1368980021004869 PMC9991839 34895386

[24] Schwingshackl L , Schwedhelm C , Hoffmann G , et al. (2017) Food groups and risk of all-cause mortality: a systematic review and meta-analysis of prospective studies. Am J Clin Nutr 105, 1462–1473. 28446499 10.3945/ajcn.117.153148

[25] Alvarado ME , Garmendia ML , Acuña G , et al. (2009) Assessment of the alcohol use disorders identification test (AUDIT) to detect problem drinkers. Rev Med Chil 137, 1463–1468. 20098805

[26] World Health Organization (2020) WHO Guidelines on Physical Activity and Sedentary Behaviour. Geneva: World Health Organization.

[27] Concha-Cisternas Y , Lanuza F , Waddell H , et al. (2019) Association between adiposity levels and cognitive impairment in the Chilean older adult population. J Nutr Sci 8, e33. 31656624 10.1017/jns.2019.24 PMC6794473

[28] Consultation WHO (2000) *Obesity: Preventing and Managing the Global Epidemic. Report of a WHO Consultation. World Health Organization Technical Report Series* no. 894, i–xii, 1–253. Geneva: WHO. 11234459

[29] Pan-American Health Organization (2003) Clinical Evaluation Modules. Module 5: Nutritional Assessment of Older Adults. Washington, DC: PAHO. https:paho.org/en/publications (accessed March 2024).

[30] Lanuza F (2024) Associations between diabesity and all-cause mortality: a prospective analysis of the Chilean National Health Survey 2009–2010. Salud Publica Mex 66, 798–806. 39977168 10.21149/15520

[31] Tapsell LC , Neale EP , Satija A , et al. (2016) Foods, nutrients, and dietary patterns: interconnections and implications for dietary guidelines. Adv Nutr 7, 445–454. 27184272 10.3945/an.115.011718 PMC4863273

[32] Brlek A & Gregorič M (2023) Diet quality indices and their associations with all-cause mortality, CVD and type 2 diabetes mellitus: an umbrella review. Br J Nutr 130, 709–718. 36423897 10.1017/S0007114522003701

[33] Mente A , Dehghan M , Rangarajan S , et al. (2023) Diet, cardiovascular disease, and mortality in 80 countries. Eur Heart J 44, 2560–2579. 37414411 10.1093/eurheartj/ehad269 PMC10361015

[34] Eleftheriou D , Benetou V , Trichopoulou A , et al. (2018) Mediterranean diet and its components in relation to all-cause mortality: meta-analysis. Br J Nutr 120, 1081–1097. 30401007 10.1017/S0007114518002593

[35] Afshin A , Sur PJ , Fay KA , et al. (2019) Health effects of dietary risks in 195 countries, 1990–2017: a systematic analysis for the Global Burden of Disease Study 2017. Lancet 393, 1958–1972. 30954305 10.1016/S0140-6736(19)30041-8 PMC6899507

[36] Zheng J , Zhu T , Li F , et al. (2023) Diet quality and mortality among Chinese adults: findings from the China Health and Nutrition Survey. Nutrients 16, 94. 38201925 10.3390/nu16010094 PMC10780502

[37] Lanuza F , Zamora-Ros R , Bondonno NP , et al. (2023) Dietary polyphenols, metabolic syndrome and cardiometabolic risk factors: an observational study based on the DCH-NG subcohort. Nutr Metab Cardiovasc Dis 33, 1167–1178. 36948936 10.1016/j.numecd.2023.02.022

[38] Lanuza F , Zamora-Ros R , Hidalgo-Liberona N , et al. (2020) Wholegrain consumption and risk factors for cardiorenal metabolic diseases in Chile: a cross-sectional analysis of 2016–2017 Health National Survey. Nutrients 12, 2815. 32937937 10.3390/nu12092815 PMC7576471

[39] Aune D , Giovannucci E , Boffetta P , et al. (2017) Fruit and vegetable intake and the risk of cardiovascular disease, total cancer and all-cause mortality—a systematic review and dose-response meta-analysis of prospective studies. Int J Epidemiol 46, 1029–1056. 28338764 10.1093/ije/dyw319 PMC5837313

[40] Lanuza F , Meroño T , Zamora-Ros R , et al. (2023) Plasma metabolomic profiles of plant-based dietary indices reveal potential pathways for metabolic syndrome associations. Atherosclerosis 382, 117285. 37778133 10.1016/j.atherosclerosis.2023.117285

[41] Kim J , Wilkens LR , Haiman CA , et al. (2024) Plant-based dietary patterns and mortality from all causes, cardiovascular disease, and cancer: the Multiethnic Cohort Study. Clin Nutr 43, 1447–1453. 38703511 10.1016/j.clnu.2024.04.035

[42] Wang Y , Liu B , Han H , et al. (2024) Correction: Associations between plant-based dietary patterns, risks of type 2 diabetes, cardiovascular disease, cancer, mortality – a systematic review, meta-analysis (Nutrition Journal (2023), 22(1), 46, 10.1186/s12937-023-00877-2). Nutr J 23, 6. 37789346 10.1186/s12937-023-00877-2 PMC10548756

[43] Lim GH , Neelakantan N , Lee YQ , et al. (2024) Dietary patterns and cardiovascular diseases in Asia: a systematic review and meta-analysis. Adv Nutr 15, 100249. 39009489 10.1016/j.advnut.2024.100249 PMC11294752

[44] Delgado-Velandia M , Maroto-Rodríguez J , Ortolá R , et al. (2022) Plant-based diets and all-cause and cardiovascular mortality in a nationwide cohort in Spain. Mayo Clin Proc 97, 2005–2015. 36333014 10.1016/j.mayocp.2022.06.008

[45] Krittanawong C , Isath A , Hahn J , et al. (2021) Fish consumption and cardiovascular health: a systematic review. Am J Med 134, 713–720. 33444594 10.1016/j.amjmed.2020.12.017

[46] Rimm EB , Appel LJ , Chiuve SE , et al. (2018) Seafood long-chain *n*-3 polyunsaturated fatty acids and cardiovascular disease: a science advisory from the American Heart Association. Circulation 138, e35–e47. 29773586 10.1161/CIR.0000000000000574 PMC6903778

[47] Mazidi M , Mikhailidis DP , Sattar N , et al. (2019) Consumption of dairy product and its association with total and cause specific mortality – a population-based cohort study and meta-analysis. Clin Nutr 38, 2833–2845. 30595374 10.1016/j.clnu.2018.12.015

[48] Tutunchi H , Naghshi S , Naemi M , et al. (2023) Yogurt consumption and risk of mortality from all causes, CVD and cancer: a comprehensive systematic review and dose–response meta-analysis of cohort studies. Public Health Nutr 26, 1196–1209. 36349966 10.1017/S1368980022002385 PMC10346031

[49] Bhandari B , Liu Z , Lin S , et al. (2023) Long-term consumption of 10 food groups and cardiovascular mortality: a systematic review and dose response meta-analysis of prospective cohort studies. Adv Nutr 14, 55–63. 36811594 10.1016/j.advnut.2022.10.010 PMC10102997

[50] MINSAL (Ministry of Health (Chile)) (2023) Guías Alimentarias para Chile (Dietary Guidelines for Chile). Chile: Gobierno de Chile.

[51] Petermann-Rocha F , Zhou Z , Mathers JC , et al. (2024) Diet modifies the association between alcohol consumption and severe alcohol-related liver disease incidence. Nat Commun 15, 6880. 39128919 10.1038/s41467-024-51314-9 PMC11317484

