# Supplementary material for: Healthy eating score and all-cause mortality: prospective findings from the Chilean National Health Survey
Source: Br J Nutr. 2025 Aug 13;134(4):304–11. doi: 10.1017/S0007114525104212 (PMC12530960; doi:10.1017/S0007114525104212)
Supplement: Lanuza et al. supplementary material [file S0007114525104212sup001.docx]

**SUPPLEMENTARY TABLE 1.** Criteria for an unweight and weighted healthy eating score.

| **Food group** | **Frequency or intake of consumption** | **Score** | **Weighted** | **RR**† |
| --- | --- | --- | --- | --- |
| Seafood | < 1 time per/month or never  1 time per/week or < 3 times per/month  > 1 time per/week | 0  1  2 | 0  0  1 | 0.90 |
| Whole grains | Never  1 time per/week or 1 time per/month  ≥ 1 portion/day or every two days | 0  1  2 | 0  0  1 | 0.79 |
| Dairy products | 1 time per/month or never  Every two days or > 1 time per/week  ≥ 1 portion/day | 0  1  2 | 0  0  1 | 0.97 |
| Fruits | < 57g/day  57g-137g/day  >137g/day | 0  1  2 | 0  0  1 | 0.90 |
| Vegetable | < 69g/day  69-137 g/day  >137g/day | 0  1  2 | 0  0  1 | 0.89 |
| Legumes | < 1 time per/week or never  1 time per/week  ≥ 2 portion/week | 0  1  2 | 0  0  1 | 0.90 |
| Total score (adherence) | Low to high adherence (0 to 12) | |  |  |

†Relative risks (RR) based on servings for each food group according optimal consumption levels: fish (100g/serving) for 2 serving/day (as statistical modeling but for practical is weekly); whole grain (30g/serving) for 3 servings/day; dairy products (200g/serving) for 1 serving/day; fruit and vegetables (80g/serving) for 3 serving/day; legumes (100g/serving) for serving/day.

**SUPPLEMENTARY TABLE 2**. Associations between unweighted healthy eating score and all-cause mortality by subgroups.

|  |  | **Ref.** | **Quartile 3** | | **Quartile 2** | | **Quartile 1** | |
| --- | --- | --- | --- | --- | --- | --- | --- | --- |
| All-cause mortality | Total cases/ events | HR (95% CI) | HR (95% CI) | p-value | HR (95% CI) | p-value | HR (95% CI) | p-value |
| Age |  |  |  |  |  |  |  |  |
| <60 years | 3,590/ 47 | 1.00 (Ref.) | 1.33  (0.45-3.95) | 0.599 | 1.57  (0.71-3.45) | 0.263 | 1.55  (0.67-3.62) | 0.304 |
| ≥ 60 years | 1746/ 229 | 1.00 (Ref.) | 1.52  (1.01-2.30) | **0.042** | 1.39  (0.97-1.98) | 0.069 | 1.58  (1.08-2.30) | **0.017** |
| Sex |  |  |  |  |  |  |  |  |
| Men | 1,957/ 126 | 1.00 (Ref.) | 1.13  (0.62-2.04) | 0.688 | 1.35  (0.83-2.21) | 0.222 | 1.52  (0.92-2.52) | 0.096 |
| Women | 3,379/ 150 | 1.00 (Ref.) | 1.72  (1.04-2.87) | **0.035** | 1.43  (0.92-2.20) | 0.104 | 1.53  (0.95-2.46) | 0.080 |
| Zone of residence |  |  |  |  |  |  |  |  |
| Urban | 4,480/ 230 | 1.00 (Ref.) | 1.63  (1.08-2.47) | **0.019** | 1.60  (1.12-2.28) | **0.009** | 1.66  (1.14-2.44) | **0.008** |
| Rural | 810/ 46 | 1.00 (Ref.) | 0.94  (0.32-2.76) | 0.916 | 0.80  (0.35-1.80) | 0.593 | 1.37  (0.61-3.08) | 0.438 |
| Geographical zone (region) |  |  |  |  |  |  |  |  |
| North (I-VI) | 1,923/ 101 | 1.00 (Ref.) | 1.56  (0.84-2.90) | 0.158 | 1.58  (0.94-2.64) | 0.082 | 2.01  (1.14-3.54) | 0.015 |
| Center (VII-IX) | 1,430/ 82 | 1.00 (Ref.) | 1.08  (0.53-2.18) | 0.821 | 1.26  (0.70-2.26) | 0.434 | 1.32  (0.68-2.57) | 0.399 |
| South (X-XV) | 1,983/ 93 | 1.00 (Ref.) | 2.05  (1.01-4.16) | **0.045** | 1.41  (0.77-2.57) | 0.265 | 1.72  (0.93-3.18) | 0.084 |
| BMI (kg/m^2^)† |  |  |  |  |  |  |  |  |
| <24.9 or <27.9 | 2,290/ 97 | 1.00 (Ref.) | 1.03  (0.47-2.25) | 0.926 | 1.96  (1.11-3.44) | **0.019** | 2.82  (1.57-5.06) | **<0.001** |
| >25.0 or >28.0 | 2,838/ 136 | 1.00 (Ref.) | 1.59  (0.93-2.71) | 0.086 | 1.34  (0.84-2.12) | 0.214 | 1.22  (0.74-2.02) | 0.432 |

HR: Hazard Ratio; BMI: Body Mass Index; Analyses are presented as HR and their 95% CI. Individuals in the quartile 1 were used as the referent. Model 2: age, sex, zone of residency, educational level, lifestyles variables (alcohol consumption, tobacco status, sleep duration, physical activity and sitting time), BMI, and multimorbidity. (as appropriate by variable of each subgroup). †WHO criteria for adult or older adults, participants who were underweight were excluded due to the potential for reverse causality (n = 208).
